# Knowledge, Attitudes, and Practices Related to Dietary Supplementation, before and during the COVID-19 Pandemic: Findings from a Cross-Sectional Survey in the Lebanese Population

**DOI:** 10.3390/ijerph18168856

**Published:** 2021-08-23

**Authors:** Hala Mohsen, Nour Yazbeck, Ayoub Al-Jawaldeh, Nazih Bou Chahine, Houssein Hamieh, Youmna Mourad, Farouk Skaiki, Hassan Salame, Pascale Salameh, Maha Hoteit

**Affiliations:** 1 Faculty of Public Health, Lebanese University, Beirut 6573, Lebanon; hala.mohsen.2@st.ul.edu.lb (H.M.); nour.yazbeck.3@st.edu.lb (N.Y.); 2 World Health Organization Regional Office for the Eastern Mediterranean, Cairo 11371, Egypt; aljawaldeha@who.int; 3 Faculty of Pharmacy, Lebanese University, Beirut 6573, Lebanon; nbouchah@ul.edu.lb (N.B.C.); pascalesalameh1@hotmail.com (P.S.); 4 Lebanese Food, Drugs and Chemical Administration, Lebanese University, Beirut 6573, Lebanon; 5 Department of Vascular Surgery, Zahraa Hospital, University Medical Center, Beirut 0961, Lebanon; 6 Nehna Hadak Association, Beirut 0961, Lebanon; houssein.hamieh87@gmail.com; 7 Al Hadi Laboratory and IVF Center, Beirut 0961, Lebanon; youmna_mourad@hotmail.com; 8 AlKarim Laboratory, Saida 0961, Lebanon; alkarimlaboratory@hotmail.com; 9 Lebanese University Task Force, Lebanese University, Beirut 6573, Lebanon; hassansalame@hotmail.com; 10 INSPECT-LB (Institut National de Santé Publique, d’Épidémiologie Clinique et de Toxicologie-Liban), Beirut 0961, Lebanon; 11 Department of Primary Care and Population Health, University of Nicosia Medical School, Nicosia 2408, Cyprus

**Keywords:** dietary, supplement, knowledge, attitude, practice, COVID-19, Lebanese population

## Abstract

At the start of 2020, a new coronavirus (COVID-19) invaded the world leading to the death of 3.92 million people. Sadly, to date, no remedy has been discovered for this virus. Preventive vaccines have been under investigation, but were unavailable until December 2020. Clinical deficiencies of nutrients may increase susceptibility to infections. This knowledge may have provided an incentive for some dietary supplement (DS) manufacturers to advertise their products as COVID-19 preventatives or cures without any substantiation, targeting mainly social media fans. The objective of this research was to assess the usage, knowledge and attitudes towards dietary supplementation before and during the COVID-19 pandemic among Lebanese people. A cross-sectional study was conducted based on a convenience sample (N = 2966) and information from participants aged 18 years and above was collected about periods before and during the pandemic. Our findings showed that attitudes towards DSs changed when the pandemic emerged and people believed that DSs can improve their health and strengthen their immunity. Despite the rise in DS prescription by healthcare professionals, the prevalence of DS use decreased from 73.3% before the pandemic to 69.9% during the pandemic (*p* < 0.001). Study results declared that the weekly or the daily estimated intake had increased during the pandemic as compared to before the pandemic, from 14% to 15.6% for antioxidants (*p* = 0.014), from 35.3% to 42.1% for vitamin C (*p* < 0.001), from 35.5% to 41% for vitamin D (*p* < 0.001), from 15.2% to 17.5% for vitamin E (*p* = 0.002), and from 18.8% to 29.3% for zinc (*p* < 0.001) and other vitamins and minerals (from 9% to 10.9%, *p* < 0.001). Binary logistic regression indicated that the use of DSs during the pandemic was 4 times higher among those infected with COVID-19, 30 times higher among those who used to take DSs before pandemic, and 1.5 times higher among those who worked in the medical sector. To conclude, there is a crucial need to increase awareness among Lebanese people regarding the use of DSs.

## 1. Introduction

The year 2020 threatened the world community [1]. The source of warning emerged in Wuhan, China, where a report of symptoms resembling those of pneumonia with unknown etiology on 19 December 2019 was published [1]. Distressingly, a new strain of coronavirus was then discovered and named as coronavirus disease (COVID-19) [2]. Global spread of the virus occurred until the COVID-19 outbreak was declared as a pandemic in March 2020 by the World Health Organization (WHO) [2]. Recent updates in June 2021 show that there are approximately 33 million total infection cases, 601,506 deaths, and 66.1% of the world’s population still not vaccinated [3]. According to the world data, only 27.1% of the world’s population has received at least one dose of a COVID-19 vaccine, and 13.7% is fully vaccinated as of 20 July 2021 [2]. The virus was characterized by rapid and aggressive transmission, along with the absence of medical treatment options [2,3]. During 2020, the clinical development process for a novel preventive vaccine was still under investigation, and all countries were competing to announce the first effective vaccine to fight the novel virus. Preventive vaccines were under investigation, but were still unavailable until December 2020. [2,3]. Consequently, people tended to rely on non-pharmacological preventive measures including public health strategies such as wearing masks, washing hands, and use of sterilizing products [2,3]. However, others also tend to rely on additional protective approaches through complementary and alternative medical options such as DSs and herb-based products, which were believed to have defensive benefits against coronavirus [2,3]. In Lebanon, the Ministry of Public health confirmed the country’s first case of novel coronavirus on 21 February 2020, and the disease started to spread uncontrollably throughout the whole country [4]. Relevant data from the Lebanese Ministry of Public health showed that there were 1422 confirmed COVID-19 cases by 13 June 2020, with an incidence rate of 208 per million persons in the country [5]. Quarantine measures were then enforced at different levels of daily life among Lebanese people affecting educational and transportation sectors [4]. However, despite these implemented measures, hospitals across the country were almost completely out of beds and suffered from dramatic increase in virus cases along with a short supply of oxygen tanks and ventilators [4], added to a severe financial crisis. 

An increase in the consumption of DSs and herbal products has been observed recently in many developed and developing countries [6]. According to the United States Food and Drug Administration (FDA), a dietary supplement is a non-drug product intended to supplement the diet with one or more of the following: vitamins, minerals, herbs, and amino acids [7]. To ensure the composition, purity and scientific characteristics of these products, FDA has established good manufacturing practices (GMPs) [8]. GMPs control the addition of ingredients, the chance of contamination, and the packaging and labeling of a product [8]. Many vitamins and minerals contained in DSs were shown to have positive impact by reducing flu and influenza symptoms in previous attempts [9]. However, most of the detected benefits were reported among those who already had a deficiency in the supplemented vitamins or minerals [9]. Although, there is a lack of evidence in supporting their utility among COVID-19 patients and further clinical trials need to be investigated [9], some micronutrients are an important concern, with promising beneficial use among COVID-19 patients. For example, the evidence of the advantageous use of vitamin D among COVID-19 patients is more powerful than that for other micronutrients [10]. Recent observations showed that those who were vitamin D deficient had a 1.77 times greater significant risk of testing positive for COVID-19 [10]. Besides, an ecological study represented certain nutrients as having higher interest for COVID-19 patients, most importantly vitamins D, C, B12, and iron, associated with lower COVID-19 incidence and/or mortality [11]. Importantly, the European Food Safety Authority (EFSA) evaluated and focused on six vitamins (D, A, C, Folate, B6, B12) and four minerals (zinc, iron, copper and selenium), essential for the normal functioning of the immune system, as evidenced by previous trials, promising for COVID-19 management and future treatment protocols [11]. Regardless of these facts, sales of DSs increased, and a survey conducted in the United States (US) showed that consumers increasingly turned towards vitamins and mineral-containing products during the pandemic period [12]. Consumers in the US tended to increase their consumption of these supplements by 10–15% since the COVID-19 pandemic began and nearly 20% who were not using DSs reported an expected trend towards using supplements in the next three months [12]. Many supplement products were proven to have added benefit in many infectious diseases, irrespective of coronavirus. Data regarding COVID-19 and DSs is still scarce and any advised DS use should depend on clinical trials. The FDA is authorized to review DSs only after they are introduced to the market. These facts explain the random use of such products which is not prevalently recognized as medication, perceived rather as food supplements [13]. While DSs have proven benefits in preventing or mitigating the impacts of diseases, adverse events after their consumption were also reported [14]. More attention to the side effects of these supplements is needed especially in such pandemic periods, in which over dependence by users is probably exacerbated. However, for most food nutrients, there are no adverse effects observed because their absorption and/or excretion are perfectly regulated [14]. High consumption of fish oils may exacerbate anticoagulation and promote bleeding in patients taking anticoagulant medications [14]. Additionally, endometriosis in women was reported after consuming isoflavones with increased risk of estrogen sensitive cancers [14]. Ephedra plant is contained in many of the body weight supplements, and contributes to increasing heart rate and blood pressure [15]. Similarly, brand names of certain herbal products are worryingly popular in Lebanon despite the huge evidence-based data about reported adverse events (liver injury, jaundice, nausea, pale stool, tiredness, fatigue, and abdominal pain) [16]. Some reports included hepatitis, acute liver failure and even death among consumers [16]. Clearly, the problem of random consumption of DSs is exacerbated during the COVID-19 pandemic, when most people spend their time online [17]. This leads the DS manufacturers to follow fraudulent approaches in order to advertise their products targeting mainly the younger generation and adolescents, the main users of social media platforms [17].Of course, consumers should know that DSs are not totally dangerous, and they can be lifesavings in situations like anemia and hypovitaminosis related conditions [17]. However, the risk begins when unnecessary amounts of these products are consumed and when they are employed to replace food in the diet [18]. The need for our research study emerged due to the observed exacerbation of the random and unnecessary consumption of supplement products, during the COVID-19 pandemic, among Lebanese people. The main goal of this research study is to assess the usage, knowledge and attitudes towards dietary supplementation before and during the COVID-19 pandemic in Lebanon. To the best of our knowledge, it the first study of its kind in Lebanon.

## 2. Materials and Methods

### 2.1. Study Design and Survey Instrument

A cross-sectional study using an online survey was conducted in Lebanon between 12 January and 21 February 2021, when the country was tragically attacked by the novel coronavirus. The World Health Organization (WHO) stated that by 1 February 2021, the country had 531 total deaths due to COVID-19 [2]. The questionnaire included 83 questions, divided into 12 major sections. The first section contained eleven questions covering demographic and socio-economic data (age, gender, residency, education, job nature, monthly income). The second section included eight questions concerning diet and health including current diet, history of diseases, COVID-19 infection status, medications taken while being infected. Sections 3 and 4 (28 questions) covered the knowledge, attitudes and practices related to DSs, and the symptoms after supplement intake “if any”. The fifth and sixth sections (6 questions) asked about the estimated intake and the reason for use of vitamins and minerals. Sections 7 and 8 (14 questions) focused on protein supplements use, estimated intake, reason for use and brand names. Sections 9 and 10 (ten questions) covered the same information regarding herbal products. The last two sections (six questions) asked about energy drinks use, estimated intake and brand names. All questions were reported at two different periods, before and during the COVID-19 pandemic. The questionnaire was self-administered and estimated to be completed by 10–15 min in the pilot testing. This questionnaire is available upon request.

### 2.2. Sampling Strategy

Eligible participants (Lebanese) were invited to participate in the survey through social media (WhatsApp, Facebook, Instagram, radio channels). It was chiefly an online survey due to forced COVID-19 pandemic lockdown restrictions and inability to conduct face to face interviews. All participants voluntarily participated in the study, and exposed to the study aims and objectives before filling the survey. The inclusion criteria were: (1) participants aged 18 years old and above; (2) Lebanese and currently residing in Lebanon. Participants were excluded if: (1) younger than 18 years old; (2) not Lebanese or currently residing outside Lebanon.

### 2.3. Sample Size

The final sample size to be included in the analysis is 2966 participants.

### 2.4. Statistical Analysis

Data were analyzed using SPSS software, version 25.0. Continuous variables were reported as mean (standard deviation (SD)), while categorical variables were reported as frequencies and percentages. The chi square test was used to determine the association between categorical variables, and the McNemar test was used to investigate the differences between categorical variables before and during the COVID-19 pandemic. Additionally, significant predictors of dietary supplement intake before and during the pandemic period were determined using binary logistic regression analysis, presented by models 1 and 2. As regards model 1, fifteen predictor variables were initially entered to the model without adjustment. However, the backwards stepwise analysis method provides the finest model to include the most significant variables in a lean representation. Backwards stepwise method lessens the variables to ten, so they are the most significant variables for estimation of the likely intake of DSs before the COVID-19 pandemic among study participants. Similarly, 17 predictor variables were initially entered to model 2 without adjustment. However, the backwards stepwise analysis method gives the finest model for including the most significant variables in a lean representation. Backwards stepwise method lessens the variables to eight, so they are the most significant variables for estimating the likely intake of DSs during the COVID-19 pandemic among study participants. The removal of the variable in the stepwise probability in the backward stepwise method was at 0.05 significance level. A confidence interval of 95% was applied, and the level of significance was predetermined at 5% (*p* < 0.05 was considered to be significant).

### 2.5. Ethical Aspects

The study was conducted according to the guidelines of the Declaration of Helsinki, and approved by the Ethics Committee of the Al-Zahraa University Medical Center, Beirut, Lebanon, reference Nb 9-2020. Anonymity of respondents was guaranteed throughout the process of data collection and analysis. Informed consent was obtained from all subjects involved in the study.

## 3. Results

### 3.1. Characteristics of Study Participants

The survey was completed by 3017 participants, and those who were less than 18 years old were excluded to end up with a sample of 2966 participants. Females represented 51.2% (n = 1522) of the study population, while 48.8% (n = 1449) were males. The average mean age of the overall sample population was 29.47 (SD = 11.4), males (M = 26.13; SD = 4.6), and females (M = 23.75; SD = 5.83). Around 47.3% of participants (n = 1402) were between 18–24 years old. In total, 39.3% of males were between 18–24 years old, while 54.9% of females were in this age group, *p* < 0.001. Nearly half of participants (50.8%, n = 1505) had normal self-reported body mass index (BMI), 30.9% (n = 915) were overweight, 12.9% (n = 384) and 5.4% (n = 161) were obese and underweight, respectively. Specifically, 40.9% of males had normal BMI, while more than half of females (60.1%) had acceptable body weight. In addition, the proportion of males with overweight (39.5%), and obesity (17.8%) was higher than females (22.7% and 8.3%, respectively), *p* < 0.001. Moreover, half of the study participants (50%, n = 1485) were residing in Beirut and the Mount Lebanon area, 19.1% (n = 568) and 19% (n = 565) were residing in North Lebanon and South Lebanon, respectively, while 11.9% (n = 354) lived in the Bekaa region. Distinctively, there was no significant difference between males and females regarding their area of residency, as nearly half of male and female participants were living in Beirut and Mount Lebanon area, *p* = 0.965. In addition, more than half of participants (63.9%, n = 1898) were single, 33.4% (n = 992) were married, while 2.7% (n = 81) were either divorced or widowed. The proportion of single females (65.9%) was significantly higher than that of males (61.8%), *p* < 0.001. Furthermore, the majority (79.2%, n = 2355) of study participants were studying or had studied at university, whereas 5.5% reported to have less than high school education. Results indicated that females who had studied or were studying at university were significantly higher in number than males who reported the same educational level (83.5% vs. 74.8%, respectively), *p* < 0.001. In total, 47.7% (n = 1416) reported having no job, 41.7% (n = 1239) and 10.6% (n = 316) had jobs in non-medical and medical sectors, respectively. Unemployed females (59.4%) were significantly higher in number than unemployed males (35.4%), *p* < 0.001. Concerning monthly income, 39.3% (n = 1167) of participants were earning 1,500,000–3,000,000 L.L, 36.4% (n = 1081) were earning < 1,500,000 L.L., and the remaining (24.3%, n = 723) admitted to earning > 3,000,000 L.L. Furthermore, lower monthly income (<1500000 L.L.) was observed more in females as compared to males (34.5% vs. 38.1%, respectively), whereas the highest monthly income (>3,000,000 L.L.) was reported more by males (28.8%), as compared to females (20.2%), *p* < 0.001. When asked about the impact of COVID-19 confinement period on the monthly income of the participants, 52.4% admitted that their income had declined. Approximately equal proportions of male and female participants admitted that their monthly income declined during the COVID-19 confinement period, *p* = 0.169. These findings are presented in Table 1.

Table 2 shows that 15.8% (n = 469) of the study population reported that they had a history of chronic diseases. Approximately an equal proportion of males and females had one or more chronic diseases, *p* = 0.528. Among the 469 sample participants who had chronic diseases, 23.6% (n = 136) had hypertension, followed by asthma (15.1%, n = 87) and diabetes (12.6%, n = 72). Other diseases were also reported including cardiovascular diseases (10.1%, n = 58), chronic psychological disorders (6.9%, n = 39), osteoporosis (3.4%, n = 20), renal diseases (1.6%, n = 9), and neoplasms (0.4%, n = 2). Moreover, hypertension was the most reported disease by males; however, asthma was mostly reported by females, *p* < 0.001. When asked about their current food regimen, 18.5% (n = 728) reported not following any specific diet, 17.9% (n = 704) were following diet for weight loss, and 16.7% (n = 659) and 12.2% (n = 479) evaluated their diets to be low in fat and salt respectively. However, others reported following high protein (9.2%, n = 364), low carbohydrate (9.8%, n = 386), high fat (2.6%, n = 103), vegetarian (4.9%, n = 195) and gluten free (2%, n = 79) diets. Another 5.7% (n = 225) were following diet for weight loss, and 0.2% (n = 8) were adhering to the intermittent fasting protocol. Moreover, among the 0.3% (n = 10) of participants who reported to be on therapeutic diets were diets to manage diabetic, Crone’s, or GERD symptoms, etc. Interestingly, a significantly higher proportion of female participants (11.3%) were following weight loss diet, as compared to 6.5% of male participants, *p* < 0.001.

Furthermore, when asked about their COVID-19 infection status, more than half (63.4%, n = 1885) stated that they were not infected, 21.7% (n = 645) were infected, and 14.9% (n = 441) were not sure about their infection status. Results showed that more males (24.3%) had been infected by the novel coronavirus, as compared to females (19.2%), *p* < 0.001. Among those who reported taking medications and supplements when they were infected, the majority reported taking supplements including vitamin C (21.3%, n = 635), followed by zinc (18.5%, n = 552) and vitamin D (17.8%, n = 529); however, analgesics like Paracetamol were the most followed medical therapy by 11.3% (n = 335) of the participants. Others stated using antibiotics (4.6%, n = 137), steroids (1.5%, n = 43), antivirals (0.2%, n = 6), and plasma (0.3%, n = 8), whereas 24.6% (n = 730) reported that they did not follow any therapy during their infectious period. Results also showed that more males (14.4%) reported not using any medical therapy, as compared to females (10.2%), *p* < 0.001.

### 3.2. Participants’ Knowledge of DSs

**Figure 1:**
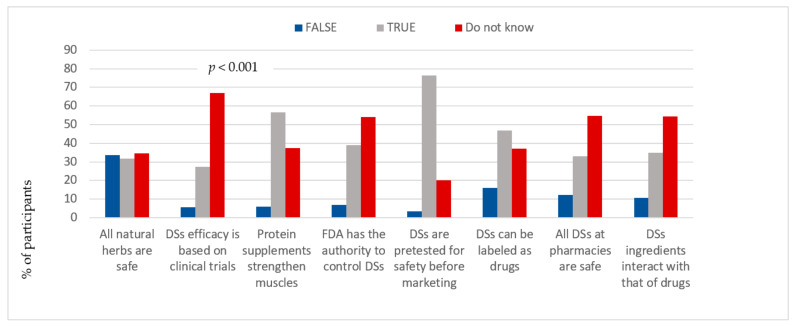
Responses of study participants to the DSs knowledge questions.

The statement that all-natural herbs are safe was approved by 31.6% (n = 939) of the participants, while 34.5% (n = 1024) had no idea concerning their safety (Figure 1). More males (37.3%) reported having no knowledge concerning this issue, as compared to females (31.9%), *p* < 0.001 (Table S1). More than half of participants (67.1%, n = 1982) reported that they did not know if the efficacy of DSs was based on clinical trials while only 27.3% (n = 805) said that they did (Figure 1). No significant difference was observed between males and females regarding the latest knowledge area, and more than half of males and females did not know the answer, *p* = 0.524 (Table S1).

Additionally, more than half of participants (56.7%, n = 1680) agreed with the claim that protein supplements can strengthen muscles, whereas 37.3% (n = 1105) seemed to have no idea concerning this effect (Figure 1). The percentage of males who approved the efficacy of protein supplements for muscle strength was significantly higher than that of females, (69.7% and 53.8%, respectively), *p* < 0.001 (Table S1).

Additionally, 38.9% (n = 1151) of respondents acknowledged that FDA regulates the safety of DSs, while more than half (54.2%, n = 1601) did not know if the FDA has this authority (Figure 1). Findings showed that more males (56.8%) reported not being familiar with the FDA role, as compared to females (51.7%), *p* = 0.023 (Table S1). Besides, 35% (n = 1033) of respondents believed that DSs can interact with drugs, while more than half (54.3%, n = 1604) did not know if supplement ingredients can interact with drugs (Figure 1). In addition, a higher level of knowledge was reported by males (55.9%), as compared to females (52.7%), when asked about possible supplement–drug interactions, *p* = 0.025 (Table S1). The majority (76.3%, n = 2261) concurred that all DSs are pretested for safety, while 20.2% (n = 597) claimed to have no idea, and 25% of males reported that they did not know this answer, as compared to 15.6% of females, *p* < 0.001 (Figure 1 and Table S1). Nearly half of participants (46.9%, n = 1389) accepted that DSs can be labeled as drugs, and 37.2% (n = 1100) seemed to have no idea about legal label requirements for DSs. Findings also showed that males had lower awareness about the legal label requirements of DSs, as compared to females, (40.9% vs. 33.5%, respectively), *p <* 0.001. Moreover, 33% (n = 973) of participants reported that supplement products available at pharmacies are safe, while more than half (54.7%, n = 1614) did not know the answer. More males (57.4%) had no answer as regards the safety of the DSs at pharmacies than females (52.1%), *p* = 0.013 (Figure 1 and Table S1).

When participants were asked about their source of advice, 54.9% and 58.9% reported using supplement products based on medical prescription before and during the COVID-19 pandemic, respectively. Findings showed that respondents relied more on dietitian’s advice (19.1%, n = 249) before the COVID-19 pandemic as compared to during the pandemic period (17.6%, n = 249). Similarly, those who used to follow the advice of sports trainers, athletes, and friends had decreased by 4.4%, 0.6% and 1.4%, respectively, during the pandemic period as compared to before that period. Furthermore, a higher significant proportion of females (76.7% vs. 77.9%) mentioned following medical prescription for DSs, as compared to males (40.5% vs. 53.1%), at both study periods (before vs. during pandemic) (*p* < 0.001) (Table S1).

Additionally, participants were asked about the source of information they sought concerning DSs Overall, health care providers were the main source of information reported to be sought by the majority (61.4% and 63.6% of participants before and during the COVID-19 pandemic, respectively). Besides, the percentage of participants who reported getting information from a friend, TV, sport trainers, supplement store salesperson, and mass media decreased by 0.7%, 0.1%, 3.4%, 0.8% and 0.6% during the COVID-19 pandemic, respectively (*p* < 0.001). However, the percentage of those who reported getting their information from trusted journals, books, and family members increased by 2.8%, 0.3% and 0.7% during the pandemic, respectively (*p* < 0.001). Furthermore, a higher significant proportion of females (69.4% vs. 63.9%) mentioned seeking information from health care providers, as compared to males (51.7% vs. 63.2%), at both study periods, before and during pandemic, respectively (Table S1).

### 3.3. Attitudes Regarding DSs Beneficial Use

**Figure 2:**
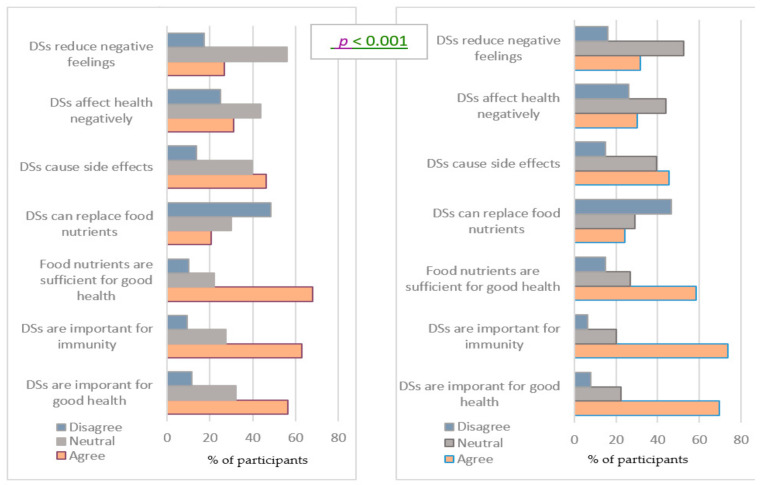
Attitudes towards DSs beneficial use, before and during COVID-19 pandemic.

Attitudes to DSs were molded during the COVID-19 pandemic period in different dimensions. Findings revealed that the pandemic significantly changed the attitude of the participants towards the importance of DSs in supporting good health, the percentage of agreement concerning this statement increasing from 56.5% before the pandemic to 69.7% during the pandemic (*p* < 0.001) (Figure 2). The proportion of females who approved this potentiality was significantly higher than that of males, before and during the pandemic period (*p* = 0.012 and *p* = 0.01, respectively). (Table S2) Similarly, the number of participants who believed that DSs are important for their immunity had increased significantly from 63% before the pandemic to 73.7% during the pandemic (*p* < 0.001) (Figure 2). The proportion of females who approved the potentiality of DSs in strengthening immunity was significantly higher than males, before and during pandemic period (*p* < 0.001 and *p* = 0.037, respectively) (Table S2). Results showed a significant decrease in the percentage of participants who reported that food nutrients are sufficient enough to support good health during the pandemic (58.5%) as compared to before the pandemic (68%) (*p* < 0.001) (Figure 2). Before the pandemic, there was no significant difference in the proportion of male and female participants who approved (*p* = 0.074), while during the pandemic a higher proportion of males (62.0%) than females (55.1%) approved the sufficiency of food nutrients, *p* < 0.001 (Table S2). The percentage of those who assumed that DSs can replace food nutrients had increased significantly from 20.6% to 24.3% once the COVID-19 pandemic emerged (*p* < 0.001). More males believed in the ability of DSs to replace food nutrients as compared to females, at both study periods (*p* < 0.001). When asked if DSs can cause symptoms, the percentage of participants who perceived supplements to cause symptoms decreased from 46.4% to 45.4% (*p* < 0.001). More females believed in the ability of DSs to cause symptoms as compared to males, before (*p* = 0.001) and during the pandemic (*p* = 0.006). Similarly, a significant decrease in the percentage of responders who believed that DSs can affect health negatively was reported, as the agreement with this statement decreased from 31.1% to 30.1% during the pandemic (*p* = 0.007). However, there was no significant difference in the proportion of males and females who perceived the ability of DSs top affect their health negatively, before the pandemic (*p* = 0.054) and during the pandemic (*p* = 0.101). Contrarily, the findings showed no significant change in the attitude of the participants concerning the ability of vitamin C supplements in protecting them from flu, the percentage of participants who approved the statement decreasing from 21.6% before the pandemic to 21.3% during the pandemic (*p* = 0.391). Similarly, there was no significant difference in the proportion of male and female participants who perceived the latest potentiality of vitamin C, at both study periods (Annex 2).

### 3.4. Practices Related to DS Use

**Figure 3:**
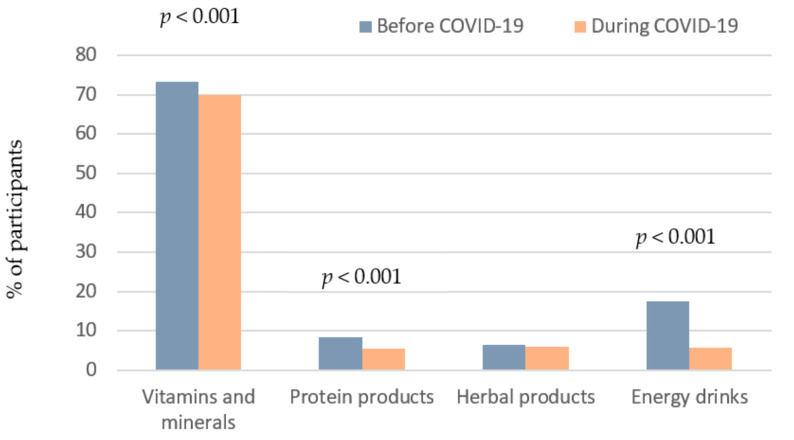
Practices related to the use of vitamins, minerals, protein products, herbal products and energy drinks before and during COVID-19 pandemic.

With regards to vitamins and minerals supplementary products, study findings revealed that the percentage of participants who used to take DSs (vitamins, minerals) before the pandemic was 73.3% (n = 2177), and this percentage decreased significantly to 69.9% (n = 2076) during the pandemic period (*p* < 0.001) (Figure 3). Besides, females reported higher prevalent use of DSs than males, before and during COVID-19 (*p* < 0.001 and *p* = 0.004, respectively) (Table S3).

Concerning protein supplements, there was a significant decrease in the percentage of participants who reported using protein-containing supplements, from 8.3% (n = 247) before the pandemic to 5.5% (n = 163) during the pandemic. Besides, protein supplements were consumed more by male participants, as compared to females, at both study periods (*p* < 0.001) (Table S3). With respect to herbal products, no significant change in their use was reported, where only 6.3% (n = 188) and 6% (n = 179) admitted using herbal products before and during the pandemic period, respectively (*p* = 0.478) (Figure 3). Besides, herbal products were consumed more by females, as compared to males, at both study periods (*p* < 0.001). The most used herbal products as reported by responders were curcumin, ginger, oregano oil, coconut oil, Aloe vera, anise, cumin, chamomile, honey, green tea, and garlic (Table S3).

With regards to energy drinks, a significantly lower percentage of the participants consumed such drinks during the pandemic (5.6%, n = 166) compared to before the pandemic (17.5%, n = 519), *p* < 0.001 (Figure 3). Besides, energy drinks were consumed more by male participants, as compared to females, at both study periods (*p* < 0.001) (Table S3).

Pharmacies were the main source of purchase of DSs, the percentage of participants who purchased supplements from pharmacies increasing significantly from 91.7% before the pandemic to 95.4% during the pandemic (*p* < 0.001). Interestingly, females tended to purchase supplement products from pharmacies more than males, at both study periods, *p* < 0.001 (Table S3). Pills were the most common form of DS used by the majority of the participants before and during the pandemic (72.6% and 73.7%, respectively, *p* < 0.001). Study results indicated that the COVID-19 emergency situation significantly changed the practice of the participants towards the reading of labels, as the percentage of participants who reported to read “always” increased from 29.2% before the pandemic to 34.3% during the pandemic, *p* < 0.001. Moreover, the proportion of females who always read the label was higher than males, before and during the COVID-19 pandemic (*p* < 0.001 and *p* = 0.016, respectively) (Table S3).

Among the 653 participants who reported avoiding DSs, 25% (n = 163) and 24.1% (n = 145) admitted that stomach pain was the main cause of avoidance in the pre pandemic period and during the pandemic period, respectively (*p* < 0.001). Besides, the high price of the DSs was represented to be another contributing factor as reported by 20.4% (n = 133) and 24.8% (n = 150) of the participants before and during pandemic, respectively (*p* < 0.001) (Table S3).

### 3.5. Estimation of Use

**Figure 4:**
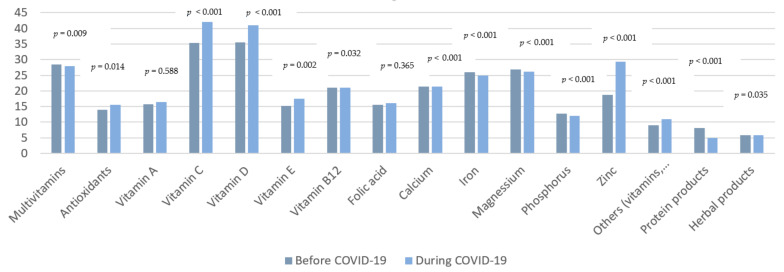
Comparison between the weekly/daily use of DSs before and during COVID-19 pandemic.

The estimated intakes of DSs were ranked as weekly or daily, monthly, or no use at all (never) (Figure 4). The weekly or the daily estimated intake of antioxidants, vitamin C, vitamin D, vitamin E, zinc, other vitamins and minerals had increased significantly during the pandemic period. However, the weekly or the daily estimated intake of multivitamins, iron, and magnesium had decreased significantly during the pandemic period, from 28.5% to 28% for multivitamins (*p* = 0.009), from 26% to 25% for iron (*p* < 0.001) and from 26.9% to 26.1% for magnesium (*p* < 0.001). The percentage of participants who stated that they never take vitamin B12 supplements had increased significantly from 69.3% before the pandemic to 70.5% during the pandemic (*p* = 0.032). Similar results for calcium were also investigated, and the percentage of participants who stated that they never take calcium supplements increased significantly from 69% beforethe pandemic to 70.8% during the pandemic (*p* < 0.001), although findings revealed no significant change in the estimated use of vitamin A (*p* = 0.588), folate (*p* = 0.365) and phosphorus (*p* = 0.576) supplements during the pandemic period as compared to before that period. Concerning protein supplements, the weekly or the daily intake of these supplements decreased significantly from 8.2% before the pandemic to 4.9% during the pandemic, while their monthly intake increased from 0.2% to 0.5% (*p* < 0.001). Similarly, when given a second option of recording protein supplements, the weekly or daily intake of these supplements decreased significantly from 2.2% before the pandemic to 1.1% during the pandemic, while the monthly intake increased from 0% to 0.1%, respectively (*p* < 0.001). Concerning herbal products, there was a significant increase in the percentage of participants who reported not using these supplements on any days of the week during the pandemic (94%) as compared to before the pandemic (93.7%), *p* = 0.035. As regards energy drinks, 99.9% of participants admitted consuming less than 7 cans/day before and during the pandemic with no significant difference (*p* = 1) (Table S4).

### 3.6. Symptoms and Adverse Events

Study participants were asked to report any symptom or adverse event they had experienced, after the use of any of the supplement products. Among those who reported using DSs, 86.9% reported that they did not feel symptoms, and 13.1% that they did. The most frequent symptom reported by 38.1% of the 256 participants was stomach pain, followed by nausea (15.5%), tachycardia (12.9%), dizziness and fatigue (12%), and headache (11.6%). Other symptoms reported by the minority were insomnia (0.9%), tremors (2.8%), tingling of the hands and legs (1.7%), gastric distress (2.8%), loss of consciousness (0.6%), and allergic and skin sensitivity reactions (1.1%).

### 3.7. Determinants of DS Intake before and during COVID-19 Pandemic Period, the Binary Logistic Analysis

The binary logistic analysis shows the relationship between the predictors of DS intake among the study population before and during COVID-19 presented as model 1 (Table 3) (before COVID-19 pandemic) and model 2 (during COVID-19 pandemic). The backward stepwise analysis of model 1 (Table 3) (before pandemic period) shows that females were more likely to use DSs as compared to males (OR = 1.793; 95% CI (1.489–2.161). Besides, those who were more educated had a higher probability of using DSs. Participants who reported studying at university were predicted to use DSs more compared to those who had lower than high school education (OR = 1.697; 95% CI (1.159–2.485). The residency was shown to be an additional determinant; the highest estimated probability of DSs intake was among those who reside in South Lebanon (OR = 1.453, 95% CI (1.129–1.870) compared to those who reside in Beirut or Mount Lebanon. Additionally, the likelihood intake of DSs is the highest among those who were employed in the non-medical sector (OR = 1.697, 95% CI (1.388–2.075) compared to those who had no job. The health status of participants was significantly associated with DSs in model 1 (Table 3), and those who reported any chronic diseases were at greater risk of using DSs compared to those who are disease free (OR = 1.69, 95% CI (1.294–2.208). Participants who did not agree with the claim announcing that DSs are important for good health were less likely to use supplements compared to those who approved this statement (OR = 0.405, 95%CI (0.281–0.582 Similarly, those who did not agree with the claim that DSs are important for their immunity were less likely to use supplements compared to those who approved this statement (OR = 0.434, 95% CI 0.296, 0.635). The backward analysis also shows that the likelihood of DS use decreases among those who have a neutral attitude towards the idea that supplements can replace food nutrients as compared to those who support this attitude (OR = 0.558, 95% CI (0.426–0.729)). Model 1 in Table 3 also highlighted the issue that trust in the label and the safety of DSs can mold the practice of their intake, and participants who admitted having no confidence in the label and the safety were less likely to use supplements (OR = 0.658, 95%CI (0.501–0.865), OR = 0.662, 95% CI (0.505–0.867) respectively). The backward stepwise analysis of model 2 (Table 4) (during the pandemic period) showed that the likelihood of DS intake increases highest among those who were employed in the medical sector as compared to those who had no job (OR = 1.697, 95% CI (1.252–2.691)). COVID-19 infection status also appeared to be a critical predictor factor; the highest intake of DSs was among those who reported that they had the virus compared to those who reported not being infected with COVID-19 (OR = 4.013, 95% CI (2.893–5.566)). Alongside model 1 (Table 3), this model also showed that the attitude of the participants concerning multiple aspects of DSs had affected their practice regarding DS intake. Participants who had a neutral response towards the claim that DSs are important for good health were less likely to use supplements compared to those who approved this statement (OR = 0.588, 95% CI (0.455–0.76)). Model 2 (Table 4) also highlighted that the participants who believed that food nutrients can be sufficient enough to support good health were less likely to use DSs, and the likelihood of DS intake increased among those who expressed disagreement towards the sufficiency of food nutrients in diet to support health and wellness, compared to those who agreed that food nutrients can be sufficient (OR = 1.79, 95%CI (1.259–2.544)). Moreover, participants who did not perceive the tendency of DSs to affect their health negatively were more likely to use these supplements compared to those who agreed with their tendency to do so (OR = 1.556, 95% CI (1.13–2.142)). Model 2 also investigated that the participants who were assured of the ability of vitamin C to protect from flu and of the safety of DSs were more likely to rely on them (OR = 1.683, 95% CI( 1.295–2.186), OR = 0.611, 95% CI ( 0.471–0.792) respectively). Participants who used to rely on DSs before the COVID-19 pandemic were very likely to persist in this practice during the pandemic (OR = 29.01, 95% CI (22.852–36.827)).

## 4. Discussion

This study explored the attitudes and knowledge with regards to different dimensions of DSs among Lebanese people. It also examined the practices related to the use of different supplement products. Our survey findings were collected and interpreted at two different periods, before and during the beginning of the COVID-19 pandemic in Lebanon. This allowed recording of the detected differences in participant’s attitudes, awareness and behaviors after COVID-19 spread across the country. Nearly half of the participants (47.3%) were between 18–24 years old, because the survey was distributed via social media platforms and Facebook groups, so most of their users are in this age group. This observation also explains the fact that the majority of our participants were single (63.9%) and studied at university (79.2%). Besides, 58.2% and 53.6% evaluated their health status and food choices as being good, respectively. However, these evaluations are based on their own discernment and not necessarily accurate. A food regimen for weight loss was reported to be most followed by respondents (17.9%). This is considered unsurprising, as the literature partly justifies our results by indicating that a perceived increase in body weight, sedentary lifestyle, unhealthy eating patterns, screen time, physical inactivity, along with fearful concerns about body weight and shape, were observed since the COVID-19 confinement period began in the USA [19]. Similar findings were observed in Poland, where 30% and over 18% of the study population reported weight gain (mean ± SD 3.0 ± 1.6 kg) and weight loss (−2.9 ± 1.5 kg), respectively, during the COVID-19 quarantine period [20]. Besides, increased BMI was associated with less frequent consumption of vegetables, fruit, and legumes, and higher consumption of meat, dairy, and fast-foods [20]. A COVID-19 Italian online survey had explored the significant change in eating habits among the Italian population, the adherence to the consumption of comfort food, and eating in response to emotions [21]. The Italian population also perceived themselves to gain weight during the COVID-19 pandemic as reported by 48.6% of the study population in another cross-sectional investigation, with a higher adherence to the Mediterranean diet [22]. Furthermore, data collected from 820 adolescents from Spain, Italy, Brazil, Colombia and Chile highlighted a modified consumption of fried food, sweet food, legumes, vegetables, and fruits among these populations, during the pandemic [23]. When asked about the therapy followed by those who admitted that they had been infected with coronavirus, more than half of the participants (57.6%) reported that they used either vitamin C or vitamin D or zinc supplement products. The highest percentage was for those who took a vitamin C supplement during their infectious period. This is due to the fact that vitamin C appeared to affect pneumonia in previous trials [24]. Particularly, three controlled trials reported a significantly lower incidence of pneumonia in vitamin C supplemented groups, suggesting its role in reducing susceptibility to lower respiratory tract infections and promoting faster relief [24]. Similarly, a small trial from India observed that patients with mild or asymptomatic COVID-19 were more likely to test negative at 21 days following daily vitamin D supplementation [25]. In addition, other observations highlighted the association between vitamin D and greater incidence or severity of SARS-CoV-2 [25]. The percentage of participants who believed that DSs are important for good health and to support their immunity increased significantly during the pandemic, but COVID-19 also reduced the trust of the general population in the ability of food nutrients to support health sufficiently. Our findings showed a decrease in the percentage of participants who reported that food nutrients are sufficient enough to support good health during the pandemic (58.5%) as compared to before (68%) (*p* < 0.001). In parallel with the latest observations, our study findings showed that the COVID-19 pandemic had caused a 4.3% significant increase in the percentage of those who trusted the ability of DSs in replacing food in their diet. Interestingly, our study investigations confirmed the results of previous studies conducted in the Middle East region. A study conducted in Saudi Arabia highlighted the popularity of the use of ginger, onion and garlic among the general population due to a belief in their ability to strengthen immunity and mitigate the chance of developing COVID-19 among the Saudi population [26]. A Saudi Arabian survey also indicated that feelings of worry, panic and misgiving in such medical emergency situations can lead to heavy dependence on alternative therapies like medicinal plants when drug treatments are not yet available [26]. Interestingly, as learned from experiences with SARS, confinement periods are known to increase the prevalence of psychological distress, such as low mood and irritability [27]. A cross-sectional study aiming to characterize the lifestyle habits, anxiety levels and basic psychological needs (BPN), in Portuguese adults during the COVID-19 pandemic showed that higher anxiety scores were observed more among females and older age groups [28]. Thus, pandemic-related quarantine is a stressful event, affecting psychological and mental health negatively, leading to post-traumatic stress symptoms, and consequent problematic eating and lifestyle behaviors, such as dietary supplementation [29]. Besides, it is important to mention that these psychological disorders were mainly responsible for eating habit changes during COVID-19 confinement [29]. A large number of UK participants (56%) reported snacking more frequently [30]. Besides, they experienced barriers to weight management compared to before lockdown, mostly among those with higher BMI [30]. Furthermore, the perceived lack of nutrients in the diet was the main reason reported by college students for taking DSs in a survey conducted in Tennessee [31]. Moreover, a previous study conducted in Lebanon also supported our latest findings, when a common attitude among Lebanese that herbal products are safer than prescription medications and of good quality was investigated [32]. Lebanese consumers were represented to have incorrect attitudes that food supplements and herbal products caused no risk and can be safely consumed [32]. Concerning knowledge, our study findings reported that 34.5% of the study population lack knowledge concerning the safety of herbal products and more than half of participants (67.1%) reported that they do not know if DSs are based on clinical trials. Additionally, 54.2% and 54.3% of participants had no conception of whether the FDA regulates the safety of DSs, and if DSs can interact with drugs, respectively. The majority (76.3%) concurred that all DSs are pretested for safety. Nearly half of the participants (46.9%) accepted that DSs can be labeled as drugs, and 54.7% reported no idea if the supplement products available at pharmacies are all considered safe. These findings are complementary with previous results in a survey employed to assess the knowledge of Lebanese people concerning DSs, in which 15% of males and 29.2% of females acknowledged that vitamins and mineral supplements do not threaten public health [32]. To wrap up, our study results revealed an alarmingly deficient knowledge of supplement’s safety, quality control, label legal requirements and interactions. Although, one of the suggested interventional methods regarding awareness related to supplementation was highlighted when a single lecture-based educational intervention was provided to college students, the good news is that the knowledge of the students concerning the safety of these products improved notably, despite only one educational session [33]. In addition to the necessary awareness, a nutri-vigilance policy is crucial in responding to side effects reported by consumers, and this will improve consumer safety by rapidly identifying possible adverse events. In Lebanon as in other countries, the market development of dietary supplements should follow these steps: (1) Validation of labels. (2) Validation of claims. (3) Validation of the formulations. (4) Dossier preparation. (5) Registration of products. (6) “Nutri-vigilance” of the product.

Additionally, our data analysis showed that when participants asked about source of advice, more than half of the responders reported using supplements based on medical prescription before and during the pandemic. Health care providers were the main source of information sought by the majority of the responders before and during the pandemic. Pharmacies were the main source of purchase of DSs in both periods. These findings partly run alongside other study results which indicated that more than half of the population (64%) reported purchasing supplements from pharmacies [32]. However, 39.5% reported that they consume DSs based on a recommendation from friend, family member or relatives, and only 24.8% upon physician’s prescription [32]. DSs should be used only after physicians’ advice; those who are iron or vitamin B12 deficient, for example, could benefit from supplementation with these vitamins and minerals [34]. Currently, the health trend is that people have adapted to take supplements on their own [34]. Study findings also showed that more than half of the study participants do not trust the safety and the label claims of the supplement products available at market (68.1% and 69.7% of participants, respectively). Research mentioned that more than half of the herbal and DSs had been approved to contain ingredients different from their label claims [35]. Additionally, more than 20% of liver damage cases reported to the United States (U.S.) Drug-Induced Liver Injury Network were caused by herbal and DSs [35]. Despite the significant changes in the attitudes of the study population towards the efficacy and the utility of DSs during such emergency periods, study findings showed a significant decrease in the percentage of the participants who took supplement products during the pandemic as compared to before that period; this could be related to the overall financial crises of the country. Similarly, a significant decrease in the percentage of those who reported using protein-based products during the pandemic has been reported. No significant change in the use of herbal products has been investigated. Study findings showed that more than half of study participants had reported using DSs before and during the pandemic period, which is considered salient, although study findings showed that the significant increase in the attitudes of participants concerning the additional beneficial effects of supplement products during COVID-19 pandemic was not accompanied with a simultaneous increase in their consumption. This can be explained by a series of events that occurred in Lebanon simultaneously with the COVID-19 emergency situation. By October 2019, the Lebanese economy was facing extraordinary challenges, capital inflows came to a sudden decrease, a black market emerged and the Lebanese Lira seemed to have no value [36]. The purchasing power of Lebanese people consequently became very low [36]. Moreover, the COVID-19 crisis along with its confinement period and the Beirut port explosion on August 4 exacerbated the economic crisis leading to financial meltdown [36]. Meanwhile, it was estimated that 4 out of 10 Lebanese had no work, and half the population is under the poverty level [36]. This has been clearly represented by our study findings, in which nearly half of the study population (47.7%) reported to have no job and more than half of study participants (52.4%) admitted that their income was reduced due to COVID-19 lockdown measures. Previous assessment approaches showed that the volume of online job postings had fallen significantly since the emergence of COVID-19 related confinement measures [37]. The cumulative effects of all mentioned events eroded people’s ability to access food and other basic needs [38]. Between July and August 2020, it was estimated that 40% of households across the country could not access markets and had deteriorating purchasing power [38]. Additionally, 19% and 55% of households were reported to consume inadequate diets and inadequate amounts of food, respectively [33]. Mostly affected are those who reside in Akkar and Baalbek El Hermel [38]. When households were asked about their coping strategy to overcome this food shortage, they admitted relying on less expensive and less preferable food [38]. Importantly, the Lebanese economic crises affected not only food, but other basic needs were also in shortage [39]. Pharmaceutical supplies including medications, baby formulas and supplements had disappeared from the pharmacies [39]. Unfortunately, the shelves become empty and these products when available were sold at many times the normal price [39], while many large pharmacies reported that most pharmaceutical products were “out of stock” [39]. Lebanese consumers found difficulty in having market or financial access to DSs and other similar products during the COVID-19 pandemic period. Our study findings showed that the high price of the DSs was represented as a frequently reported contributing factor for avoiding DSs by 20.4% and 24.8% of the participants before and during the pandemic, respectively (*p* < 0.001).

Furthermore, when asked about the estimated use of a series of vitamins and minerals, a significant increase in the weekly and daily intake of antioxidants, vitamin C, vitamin D, vitamin E, calcium and zinc was observed. This is due to the belief that these nutrients had proven ability as immune-boosting, having an antiviral action, being antioxidant, and characterized by anti-inflammatory effects [40]. Consequently, combining these nutrients in the form of a DS may prevent virus spread, and provide therapeutic support against COVID-19 [40]. Additionally, the most reported reason to use DSs by our study population was to promote general health and wellbeing, before and during the pandemic. This is considered compatible with previous investigations which showed that US adults use DSs to improve (45%) and maintain (33%) overall health. Model 2 of the binary logistic regression revealed that those who reported working in medical sectors were approximately two times more likely to take DSs compared to those who had no job (*p* = 0.02). This is due to the fact that doctors, nurses and health care professionals are more in direct contact with patients in hospitals and clinical areas. Health care providers are seven times more likely to have severe COVID-19 infection [41]. Health care workers and their families represented 17% of hospital admissions for COVID-19 for those aged 18–65 years old [41]. Moreover, 1 in 6 hospital COVID-19 cases are among health care providers and their families [42]. In addition, an increase in mental health conditions (anxiety, depression, suicidal ideation) has been documented more frequently among health care workers as compared to the general population, since the start of the COVID-19 pandemic [43]. This is due to the fact that physicians and public health workers suffered from a prolonged period of psychological stress in responding to the pandemic and implementing an unprecedented vaccination campaign [43]. Those who were infected with the virus during the pandemic were also predicted to be five times more likely to use DSs as compared to those who reported not being infected (*p* < 0.001). Several studies approved these nutritional interventions acting as immunity stimulators and providing defense against viral infections [44]. Interestingly, even if food nutrients are accessible and available, the use of DS are seen as promising for the prevention or mitigation of COVID-19 related symptoms [44]. However, due to the lack of evidence and World Health Organization’s guidelines about linkage between nutrients supplementation and prevention of COVID-19 infection, there is a need to design and implement strong clinical trials to assure the utility of these supplement products among COVID-19 patients [45]. Most promising, as mentioned by the European Food Safety Authority (EFSA), are the six vitamins (D, A, C, Folate, B6, B12) and the four minerals (zinc, iron, copper and selenium) for COVID-19 management; these are shown to have more potent benefit in the prevention, treatment and management of COVID-19 symptoms [11].

A history of chronic diseases was a contributing factor in using DSs before the pandemic period, but not during the pandemic. This can be partially related to the intentional focus on these diseased patients during such infectious situations by their health care providers [46]. Additionally, most of them had more frequent conversations and consultations with their health care providers during the pandemic [46]. Consequently, their sources of information and advice regarding the use of DSs are more evidence based, and this may reduce the risk of random and unnecessary use among diseased patients. 

The main strength of our study is that a representative large sample size was recruited. However, there are some limitations. The cross-sectional design of our survey limited our ability to make a causal inference. The shortage of studies that are known to evaluate the behaviors, knowledge and practices regarding the use of DSs during the COVID-19 pandemic worldwide, and in the Middle East specifically, limited our ability to compare our findings with those of other Arabic countries. Besides, the online survey itself excluded a possible targeted vulnerable population which is difficult reach through social media platforms, leading to a probable selection bias. The study results being self-reported by the population may cause a non-differential information bias, which might bias the results towards the null. Most importantly, the economic crises in Lebanon at the time of data collection appeared to be a confounding factor for our study results.

## 5. Conclusions

Study participants appeared to have a shortage of essential knowledge concerning multiple aspects related to DS use. They also seemed to have distinct misconceptions regarding their potentiality. It is important to know that not all DSs have to be treated equally regarding COVID-19 management, as for the case of some vitamins like vitamins D, C and E which may have real benefits, unlike certain herbs which may be useless against COVID-19. The current study emphasized the crucial need to increase awareness among Lebanese people regarding the use of DSs. They should be aware about the fact that supplementary products can be used to fill in nutritional gaps in one’s diet and can be highly beneficial for those who suffer from deficiencies or are in a need of higher amounts A synergistic effort of policy makers and health care providers is necessary to guide the process of the safe use of DSs through educating, monitoring and taking necessary actions. Study results will be hopefully employed in future approaches, with the cooperation of the Lebanese Ministry of Public Health. The long term sustained plan is to explore the Nutri-vigilance policy in Lebanon, which will protect the health of the Lebanese consumer and guarantee safe use of DSs available in the Lebanese market, based on the reported side effects of Lebanese users.

## Figures and Tables

**Table 1:** Demographic and socio-economic characteristics of the study population, overall and by gender.

		Overall		Males		Females		
		Mean	SD	Mean	SD	Mean	SD	
Age		29.47	11.4	26.13	4.6	23.75	5.83	
		N	%	N	%	N	%	*p*-value
Age Categories	18–24	1402	47.3	569	39.3	833	54.9	<0.001
	>24	1560	52.7	877	60.7	683	45.1	
BMI	Underweight	161	5.4	26	1.8	135	8.9	<0.001
	Normal	1505	50.8	592	40.9	913	60.1	
	Overweight	915	30.9	571	39.5	344	22.7	
	Obese	384	12.9	258	17.8	126	8.3	
Gender	Male	1449	48.8	-	-	-	-	-
	Female	1522	51.2	-	-	-	-	-
Residency	Beirut or Mount Lebanon	1485	50.0	719	49.6	766	50.3	0.965
	South Lebanon	565	19.0	275	19.0	290	19.1	
	Bekaa	354	11.9	176	12.2	177	11.6	
	North Lebanon	568	19.1	279	19.3	289	19	
Marital Status	Single	1898	63.9	895	61.8	1003	65.9	<0.001
	Married	992	33.4	529	36.5	463	30.4	
	Others	81	2.7	26	1.8	56	3.7	
Education	Less than high school	162	5.5	89	6.1	73	4.8	<0.001
	High school	455	15.3	277	19.1	178	11.7	
	University	2355	79.2	1083	74.8	1271	83.5	
Job Nature	No Job	1416	47.7	513	35.4	904	59.4	<0.001
	Medical sector	316	10.6	160	11.0	156	10.3	
	Non-Medical sector	1239	41.7	777	53.6	462	30.4	
Monthly Income	<1,500,000 L.L.	1081	36.4	500	34.5	581	38.1	<0.001
	1,500,000 −3,000,000 L.L.	1167	39.3	532	36.7	635	41.7	
	>3,000,000 L.L.	723	24.3	417	28.8	307	20.2	
Impact of COVID-19 pandemic on monthly income	Yes	1038	34.9	484	33.4	553	36.3	0.169
	No	1556	52.4	778	53.7	778	51.1	
	¼ income	126	4.2	69	4.7	57	3.8	
	½ income	170	5.7	85	5.8	85	5.6	
	¾ income	82	2.7	34	2.3	48	3.2	

**Table 2:** Health and COVID-19 infection status of the study participants, overall and by gender.

		Overall		Males		Females		
		N	%	N	%	N	%	*p*-Value
Have Chronic disease	No	2502	84.2	1214	83.8	1288	84.6	0.528
	Yes	469	15.8	235	16.2	234	15.4	
Type of disease	Cardiovascular	58	10.1	38	6.6	20	3.5	<0.001
	Hypertension	136	23.6	86	14.9	50	8.6	
	Asthma	87	15.1	37	6.4	50	8.7	
	Renal	9	1.6	6	1.0	4	0.6	
	Neoplasms	2	0.4	0	0.0	2	0.4	
	Diabetes	72	12.6	42	7.3	31	5.3	
	Osteoporosis	20	3.4	5	0.8	15	2.6	
	Psychological Disorders	39	6.9	20	3.5	19	3.4	
	GI Tract Disorders	27	4.7	14	2.4	14	2.4	
	Thyroid Problems	30	5.3	8	1.4	23	3.9	
	Blood Disorders	29	5.1	11	1.9	18	3.1	
	Neurological Disorders	20	3.4	5	0.9	15	2.5	
	Immune or Inflammatory Diseases	31	5.4	12	2.1	19	3.3	
	Skin Problems	7	1.2	0	0.0	7	1.2	
	Joint or Back Problems	7	1.2	3	0.5	4	0.7	
Current Diet	Weight loss	704	17.9	259	6.6	445	11.3	<0.001
	Weight gain	225	5.7	133	3.4	92	2.3	
	High Protein	364	9.2	252	6.4	111	2.8	
	High Fat	103	2.6	77	1.9	26	0.7	
	Low Fat	659	16.7	330	8.4	329	8.3	
	Low Carbohydrate	386	9.8	200	5.1	186	4.7	
	Low Salt	479	12.2	261	6.6	219	5.5	
	Vegetarian	195	4.9	89	2.3	106	2.7	
	Gluten Free	79	2.0	53	1.3	26	0.7	
	Intermittent Fasting	8	0.2	1	0.0	6	0.2	
	Dairy Free	1	0.0	0	0.0	1	0.0	
	Non-Specific	728	18.5	339	8.6	388	9.8	
	Therapeutic	10	0.3	4	0.1	7	0.2	
COVID-19 infection status	Not Infected	1885	63.4	862	59.4	1024	67.2	<0.001
	Got Infected	645	21.7	352	24.3	293	19.2	
	Not Sure	441	14.9	236	16.3	206	13.5	
Medications for COVID-19 management	No medications	730	24.6	428	14.4	303	10.2	<0.001
	Vitamin C	635	21.3	318	21.8	317	21.8	
	Vitamin D	529	17.8	259	10.7	270	10.7	
	Zinc	552	18.5	281	8.7	270	9.1	
	Analgesic	335	11.3	161	9.5	174	9.1	
	Antibiotics	137	4.6	66	5.4	71	5.8	
	Steroids	43	1.5	19	0.7	25	0.8	

**Table 3:** Regression analysis, Backward LR Odds Ratio (Model 1).

Determinants of DSs Use before COVID-19 Pandemic Survey Question: Did You Use DSs? No (Reference), Yes.	OR	95% C.I. For EXP (B)		*p*-Value
		Lower	Upper	
**Gender**				
Male (Reference).	1.00			
Females as compared to males.	1.793	1.489	2.161	0.000
**Education**				
less than high school (Reference).	1.00			
High school level as compared to less than high school.	1.332	0.874	2.031	0.182
University as compared to less than high school.	1.697	1.159	2.485	0.007
**Residency**				
Beirut and Mount Lebanon (Reference).	1.00			
South Lebanon as compared to Beirut and Mount Lebanon.	1.453	1.129	1.870	0.004
Bekaa as compared to Beirut and Mount Lebanon.	1.253	0.939	1.673	0.126
North Lebanon as compared to Beirut and Mount Lebanon.	0.713	0.567	0.897	0.004
**Job Nature**				
No job (Reference).	1.00			
Medical sector as compared to having no job.	0.973	0.726	1.304	0.854
Non-Medical sector as compared to having no job.	1.697	1.388	2.075	0.000
**Chronic disease status**				
Not having CD (Reference).	1.00			
Having CD as compared to not having CD.	1.690	1.294	2.208	0.000
**DSs are important for good health**				
Agree (Reference).	1.00			
Neutral as compared to agree attitude.	0.590	0.461	0.755	0.000
Disagree as compared to agree attitude.	0.405	0.281	0.582	0.000
**DSs are important for Immunity**				
Agree (Reference).	1.00			
Neutral as compared to agree attitude.	0.694	0.543	0.887	0.003
Disagree as compared to agree attitude.	0.434	0.296	0.635	0.000
**DSs can replace food nutrients**				
Agree (Reference).	1.00			
Neutral as compared to agree attitude.	0.558	0.426	0.729	0.000
Disagree as compared to agree attitude.	0.915	0.700	1.197	0.517
**Trust of label**				
Being confident (Reference).	1.00			
Being non-confident as compared to being confident.	0.658	0.501	0.865	0.003
**Trust of safety**				
Being confident (Reference).	1.00			
Being non-confident as compared to being confident.	0.662	0.505	0.867	0.003

**Table 4:** Regression Analysis, Backward LR Odds Ratio (Model 2).

Determinants of DSs Use During COVID-19 Pandemic. Survey Question: Do you Take DSs? No (Reference), Yes	OR	95% C.I. For EXP (B)		*p*-Value
		Lower	Upper	
**Job Nature**				
No job (Reference).	1.00			
Medical sector as compared to having no job.	1.836	1.252	2.691	0.002
Non-medical sector as compared to having no job.	1.365	1.081	1.723	0.009
**COVID-19 infection status**				
Not getting the infection (Reference).	1.00			
Got infected as compared to not getting the infection.	4.013	2.893	5.566	0.000
Being not sure as compared to not getting the infection.	1.236	0.915	1.669	0.167
**DSs are important for good health**				
Agree (Reference).	1.00			
Neutral as compared to agree attitude.	0.588	0.455	0.76	0.000
Disagree as compared to agree attitude.	0.853	0.567	1.283	0.445
**Food nutrients are sufficient for good health**				
Agree (Reference).	1.00			
Neutral as compared to agree attitude.	1.133	0.876	1.465	0.341
Disagree as compared to agree attitude.	1.79	1.259	2.544	0.001
**DSs affect health negatively**				
Agree (Reference).	1.00			
Neutral as compared to agree attitude.	0.909	0.702	1.176	0.467
Disagree as compared to agree attitude.	1.556	1.131	2.142	0.007
**Vitamin C supplements protect from flu**				
No (Reference).	1.00			
Yes response as compared to no response.	1.683	1.295	2.186	0.000
**Trust of safety**				
Being confident (Reference)	1.00			
Being non-confident as compared to being confident.	0.611	0.471	0.792	0.000
**DSs use before COVID-19 pandemic**				
No (Reference)	1.00			
Yes response as compared to no response	29.01	22.852	36.827	0.000

## Data Availability

This study did not report any data yet.

## Supplementary Materials

The following are available online at https://www.mdpi.com/article/10.3390/ijerph18168856/s1, Table S1: Participants’ Knowledge about DSs by gender; Table S2: Attitudes regarding DSs beneficial use by gender; Table S3: Practices regarding DSs beneficial use by gender; Table S4: Estimated use of DSs.

## References

[1] Bogoch I.I., Watts A., Thomas-Bachli A., Huber C., Kraemer M.U., Khan K. (2020). Pneumonia of unknown aetiology in Wuhan, China: Potential for international spread via commercial air travel. J. Travel Med..

[2] World Health Organization WHO Coronavirus (COVID-19) Dashboard. https://COVID19.who.int/.

[3] Centers for Disease Control and Prevention Estimated Disease Burden of COVID-19. https://www.cdc.gov/coronavirus/2019-ncov/cases-updates/burden.html.

[4] Lebanon: First Case of Coronavirus (COVID-19) Confirmed February 21. Crisis24. https://crisis24.garda.com/fr/ressources/alertes-de-securite/qasde7jsb6oyeccse/lebanon-first-case-of-coronavirus-COVID-19-confirmed-february-21.

[5] COVID-19 Coronavirus Lebanon Cases. https://moph.gov.lb/maps/covid19.php.

[6] National Policy on Traditional Medicine and Regulation of Herbal Medicines. https://apps.who.int/iris/bitstream/handle/10665/43229/9241593237.pdf.

[7] Food and Drug Administration. https://www.fda.gov/consumers/consumer-updates/dietary-supplements.

[8] Center for Food Safety and Applied Nutrition CGMPs for Food and DSs. U.S. Food and Drug Administration. https://www.fda.gov/food/guidance-regulation-food-and-dietary-supplements/current-good-manufacturing-practices-cgmps-food-and-dietary-supplements.

[9] Kubala J. Can. Supplements Fight Coronavirus (COVID-19)? 15 Immune Boosters. Healthline. https://www.healthline.com/nutrition/immune-boosting-supplements.

[10] Meltzer D.O., Best T.J., Zhang H., Vokes T., Arora V., Solway J. (2020). Association of Vitamin D status and other clinical characteristics with COVID-19 test results. JAMA Netw. Open.

[11] Galmés S., Serra F., Palou A. (2020). Current state of evidence: Influence of nutritional and nutrigenetic factors on immunity in the covid-19 pandemic framework. Nutrients.

[12] Grebow J. Peak Dietary Supplement Sales Leveling off during COVID-19 Pandemic, but Growth Still Remains Strong over Last Year, Market Researchers Report during Webcast. Nutritional Outlook. https://www.nutritionaloutlook.com/view/peak-dietary-supplement-sales-leveling-during-COVID-19-pandemic-growth-still-remains-strong.

[13] Center for Food Safety and Applied Nutrition Questions and Answers on Dietary Supplements. U.S. Food and Drug Administration. https://www.fda.gov/food/information-consumers-using-dietary-supplements/questions-and-answers-dietary-supplements.

[14] Ronis M.J.J., Pedersen K.B., Watt J. (2018). Adverse Effects of Nutraceuticals and DSs. Annu. Rev. Pharmacol. Toxicol..

[15] The Dangers of the Herb Ephedra. Harvard Health. https://www.health.harvard.edu/staying-healthy/the-dangers-of-the-herb-ephedra.

[16] Herbalife Side Effects and Horror Stories. Sweet Science of Fighting. https://sweetscienceoffighting.com/herbalife-side-effects-and-horror-stories/.

[17] Influencers, Social Media Advertising, and Litigation Risks for Food and Dietary Supplement Companies. Food and Drug Law Institute (FDLI). https://www.fdli.org/2020/05/influencers-social-media-advertising-and-litigation-risks-for-food-and-dietary-supplement-companies/.

[18] Should You Get Your Nutrients from Food or from Supplements? Harvard Health. https://www.health.harvard.edu/staying-healthy/should-you-get-your-nutrients-from-food-or-from-supplements.

[19] Keel P.K., Gomez M.M., Harris L., Kennedy G.A., Ribeiro J., Joiner T.E. (2020). Gaining “The Quarantine 15”: Perceived versus observed weight changes in college students in the wake of COVID-19. Int. J. Eat. Disord..

[20] Sidor A., Rzymski P. (2020). Dietary Choices and Habits during COVID-19 Lockdown: Experience from Poland. Nutrients.

[21] Di Renzo L., Gualtieri P., Cinelli G., Bigioni G., Soldati L., Attinà A., Bianco F.F., Caparello G., Camodeca V., Carrano E. (2020). Psychological Aspects and Eating Habits during COVID-19 Home Confinement: Results of EHLC-COVID-19 Italian Online Survey. Nutrients.

[22] Di Renzo L., Gualtieri P., Pivari F., Soldati L., Attinà A., Cinelli G., Leggeri C., Caparello G., Barrea L., Scerbo F. (2020). Eating habits and lifestyle changes during COVID-19 lockdown: An Italian survey. J. Transl. Med..

[23] Ruiz-Roso M.B., de Carvalho Padilha P., Mantilla-Escalante D.C., Ulloa N., Brun P., Acevedo-Correa D., Arantes Ferreira Peres W., Martorell M., Aires M.T., de Oliveira Cardoso L. (2020). Covid-19 Confinement and Changes of Adolescent’s Dietary Trends in Italy, Spain, Chile, Colombia and Brazil. Nutrients.

[24] Holford P., Carr A.C., Jovic T.H., Ali S.R., Whitaker I.S., Marik P.E., Smith A.D. (2020). Vitamin C-An Adjunctive Therapy for Respiratory Infection, Sepsis and COVID-19. Nutrients.

[25] Vimaleswaran K.S., Forouhi N.G., Khunti K. Vitamin D and Covid-19. The BMJ. https://www.bmj.com/content/372/bmj.n544.

[26] Alyami H.S., Orabi M.A., Aldhabbah F.M., Alturki H.N., Aburas W.I., Alfayez A.I., Alharbi A.S., Almasuood R.A., Alsuhaibani N.A. (2020). Knowledge about COVID-19 and patients’ Attitudes about and use of herbal products during the COVID-19 pandemic: A cross-sectional study in Saudi Arabia. Saudi Pharm. J..

[27] Adhanom Ghebreyesus T. (2020). Addressing mental health needs: An integral part of COVID -19 response. World Psychiatry.

[28] Antunes R., Frontini R., Amaro N., Salvador R., Matos R., Morou√ßo P., Rebelo-Gon√ßalves R. (2020). Exploring Lifestyle Habits, Physical Activity, Anxiety and Basic Psychological Needs in a Sample of Portuguese Adults during COVID-19. Int. J. Environ. Res. Public. Health.

[29] Rodgers R.F., Lombardo C., Cerolini S., Franko D.L., Omori M., Fuller-Tyszkiewicz M., Linardon J., Courtet P., Guillaume S. (2020). The impact of the COVID -19 pandemic on eating disorder risk and symptoms. Int. J. Eat. Disord..

[30] Robinson E., Boyland E., Chisholm A., Harrold J., Maloney N.G., Marty L., Mead B.R., Noonan R., Hardman C.A. (2021). Obesity, eating behavior and physical activity during COVID-19 lockdown: A study of UK adults. Appetite.

[31] Webb A.D. Dietary Supplement Use and Attitudes among College Students Enrolled in an Introductory Nutrition Course. https://trace.tennessee.edu/utk_gradthes/67/.

[32] El Khoury G., Ramadan W., Zeeni N. (2015). Herbal Products and DSs: A Cross-Sectional Survey of Use, Attitudes, and Knowledge among the Lebanese Population. J. Community Health.

[33] Chiba T., Kobayashi E., Okura T., Sekimoto M., Mizuno H., Saito M., Umegaki K. (2020). An educational intervention improved knowledge of dietary supplements in college students. BMC Public Health.

[34] Self-Prescribing Supplements? Here’s Why You Should Think Again. Henry Ford LiveWell. https://www.henryford.com/blog/2021/01/self-prescribing-supplements.

[35] Thompson D. Can You Trust the Labels on Your Supplements?. https://www.webmd.com/vitamins-and-supplements/news/20171102/can-you-trust-the-labels-on-your-supplements.

[36] Dagher S., Majzoub A., Abi-Nassif C. Lebanon’s Economic Crisis: A Tragedy in the Making. Middle East Institute. https://www.mei.edu/publications/lebanons-economic-crisis-tragedy-making.

[37] An Assessment of the Impact of COVID-19 on Job and Skills Demand Using Online Job Vacancy Data. OECD. https://www.oecd.org/coronavirus/policy-responses/an-assessment-of-the-impact-of-COVID-19-on-job-and-skills-demand-using-online-job-vacancy-data-20fff09e/.

[38] Lebanon: M-VAM Vulnerability and Food Security Assessment July-August 2020-Lebanon. ReliefWeb. https://reliefweb.int/report/lebanon/lebanon-m-vam-vulnerability-and-food-security-assessment-july-august-2020.

[39] Limited B.P.P.C. Mid-Pandemic, Lebanon Medicine Shortages Sow Panic. https://www.bangkokpost.com/world/2064543/mid-pandemic-lebanon-medicine-shortages-sow-panic.

[40] Mrityunjaya M., Pavithra V., Neelam R., Janhavi P., Halami P.M., Ravindra P.V. (2020). Immune-Boosting, Antioxidant and Anti-inflammatory Food Supplements Targeting Pathogenesis of COVID-19. Front. Immunol..

[41] Healthcare Workers 7 Times as Likely to Have Severe COVID-19 as Other Workers. BMJ. https://www.bmj.com/company/newsroom/healthcare-workers-7-times-as-likely-to-have-severe-COVID-19-as-other-workers/.

[42] Health Workers and Their Families Account for 1 in 6. https://www.bmj.com/company/newsroom/health-workers-and-their-families-account-for-1-in-6-hospital-COVID-19-cases/.

[43] Orquiola D., Lynfield R., Shah D., Freeman L., Becker S., Williams A., Gould D.W., Tiesman H., Lloyd G., Hill L. (2021). Symptoms of Depression, Anxiety, Post-Traumatic Stress Disorder, and Suicidal Ideation among State, Tribal, Local, and Territorial Public Health Workers during the COVID-19 Pandemic-United States, March-April 2021. MMWR Morb. Mortal. Wkly. Rep..

[44] Moscatelli F., Sessa F., Valenzano A., Polito R., Monda V., Cibelli G., Villano I., Pisanelli D., Perrella M., Daniele A. (2021). COVID-19: Role of Nutrition and Supplementation. Nutrients.

[45] AbdAllah M., Ez Elarab H., Raslan E., Saber L., Daoud E., Saber M. (2020). Role of micronutrients in the management of coronavirus disease 2019. New Microbes New Infect..

[46] Chronic Disease Management during COVID-19-NCSL. https://www.ncsl.org/documents/health/Chronic-Disease-Management-During-COVID-19-webinar.pdf.

