# Supplementary material for: Knowledge, Attitudes, and Practices Related to Dietary Supplementation, before and during the COVID-19 Pandemic: Findings from a Cross-Sectional Survey in the Lebanese Population
Source: Int J Environ Res Public Health. 2021 Aug 23;18(16):8856. doi: 10.3390/ijerph18168856 (PMC8395050; doi:10.3390/ijerph18168856)
Supplement: Supplementary file 1 [file ijerph-18-08856-s001.zip › ijerph-1311545-supplementary.pdf]

ANNEX 1: Participants' Knowledge about DSs by gender

		Males		Females		
		N	%	N	%	P-value
**All-natural herbs are safe**	False	402	27.8	602	39.6	<0.001
	True	505	34.9	434	28.5	
	Do Not Know	540	37.3	484	31.9	
**DSs efficacy are based on clinical trials**	False	79	5.5	87	5.8	0.524
	True	381	26.4	424	28.1	
	Do Not Know	983	68.1	999	66.2	
**Protein supplements strengthen muscles**	False	57	3.9	123	8.1	<0.001
	True	865	69.7	815	53.8	
	Do Not Know	527	36.4	578	38.1	
**FDA controls DSs**	False	93	6.4	110	7.3	0.023
	True	531	36.8	620	41.0	
	Do Not Know	819	56.8	782	51.7	
**Supplement ingredients interact with that of drugs**	False	152	10.5	167	11.0	0.025
	True	485	33.6	548	36.3	
	Do Not Know	808	55.9	796	52.7	
**DSs are pretested for safety**	False	58	4.0	47	3.1	<0.001
	True	1028	71.0	1233	81.3	

	Do Not Know	361	25.0	236	15.6	
**DSs can be labeled as drugs**	False	235	16.3	236	15.6	<0.001
	True	618	42.8	770	50.9	
	Do Not Know	592	40.9	508	33.5	
**All supplement products in pharmacies are safe**	False	166	11.5	197	13.1	0.013
	True	448	31.0	525	34.9	
	Do Not Know	830	57.4	784	52.1	
**Source of advice before COVID-19**	Medical Prescription	202	40.5	519	76.7	<0.001
	Dietitian's Advice	130	26.1	119	17.7	
	Sport Trainer Advice	135	27.0	12	1.7	
	Athlete Advice	15	3.0	2	0.3	
	Friends Advice	18	3.5	24	3.6	
	Other Motives	0	0.0	0	0.0	
**Source of advice during COVID-19.**	Medical Prescription	306	53.1	527	77.9	<0.001
	Dietitian's Advice	150	25.9	99	14.7	
	Sport Trainer Advice	83	14.4	13	2.0	
	Athlete Advice	9	1.5	1	0.2	
	Friends Advice	29	5.1	36	5.3	
	Other Motives	0	0	0	0	
**Source of information before COVID-19.**	Health Care Providers	358	51.7	580	69.4	<0.001
	Trusted Journals	62	8.9	70	8.4	

	Sport Trainers	102	14.7	7	0.8	
	Books	1	0.2	3	0.4	
	Magazines	3	0.4	1	0.1	
	TV	1	0.2	3	0.3	
	Store Sale Person	13	1.9	9	1.0	
	Family Members	10	1.4	23	2.8	
	Friends	44	6.4	30	3.6	
	Mass Media	72	10.3	74	8.8%	
	Other	28	4.0	37	4.4	
**Source of information during COVID-19**	Health Care Providers	461	63.2	566	63.9	0.001
	Trusted Journals	74	10.2	110	12.4	
	Sport Trainers	25	3.5	35	3.9	
	Books	6	0.8	4	0.5	
	Magazines	2	0.3	1	0.1	
	TV	0	0.0	3	0.3	
	Store Sale Person	3	0.4	7	0.8	
	Family Members	32	4.4	15	1.7	
	Friends	26	3.5	43	4.8	
	Mass Media	61	8.3	82	9.3	
	Other	40	5.5	21	2.4	

ANNEX 2: Attitudes regarding DSs beneficial use by gender

		Before COVID-19 pandemic				During COVID-19 pandemic				P value	
		Male		Female		Male		Female			
		N	%	N	%	N	%	N	%	P1*	P2*
DSs are important for good health	Agree	811	56	868	57	977	67.4	1094	71.9	0.012	0.010
	Neutral	492	34	457	30.1	360	24.8	308	20.2		
	Disagree	146	10.1	196	12.9	112	7.8	121	7.9		
DSs are important for immunity	Agree	899	62	974	64	1046	72.2	1143	75.1	0.000	0.037
	Neutral	442	30.5	379	24.9	319	22	279	18.3		
	Disagree	108	7.5	169	11.1	84	5.8	100	6.5		
Food nutrients are sufficient for good health	Agree	1002	69.1	1017	66.8	899	62	838	55.1	0.074	0.000
	Neutral	322	22.2	336	22.1	367	25.3	425	27.9		
	Disagree	125	8.6	169	11.1	184	12.7	259	17		
DSs can replace the food nutrients	Agree	331	22.8	279	18.3	392	27.1	330	21.6	0.000	0.000
	Neutral	505	34.9	417	27.4	463	31.9	399	26.2		
	Disagree	613	42.3	826	54.3	594	41	793	52.1		
DSs can cause symptoms	Agree	624	43	754	49.5	625	43.1	726	47.7	0.001	0.006
	Neutral	624	43	561	36.9	614	42.4	558	36.7		
	Disagree	202	13.9	207	13.6	210	14.5	238	15.6		
DSs affect health negatively	Agree	422	29.1	504	33.1	410	28.3	485	31.9	0.054	0.101
	Neutral	661	45.6	643	42.3	658	45.4	650	42.7		
	Disagree	367	25.3	275	24.7	381	26.3	318	20.9		
Vitamin C supplement protects from flu	Yes	1124	77.6	1204	79.1	1139	78.6	1199	78.8	0.310	0.088
	No	325	22.4	318	20.9	311	21.4	323	21.2		

P1: significance value for male and female comparison before COVID-19

P2: significance value for male and female comparison during COVID-19

ANNEX 3: Practices regarding DSs beneficial use by gender

	Before COVID-19 pandemic				During COVID-19 pandemic				P value	
	Male		Female		Male		Female		P1*	P2*
	N	%	N	%	N	%	N	%		

DSs Use	Yes	999	68.9	1178	77.4	977	67.4	1100	72.2	0.000	0.004
	No	451	31.1	344	22.6	473	32.6	423	27.8		
Cause of avoidance of DSs	Medical Reason	18	12.5	22	12	18	12.9	22	12	0.001	0.003
	Stomach Pain	35	24.5	65	35.8	40	28.2	60	32.9		
	Insomnia	17	12.1	19	10.6	17	11.6	22	12.2		
	Tachycardia	15	10.8	1	0.7	15	10.8	1	0.7		
	Depressive symptoms	11	7.4	8	4.4	5	3.3	8	4.4		
	Nervousness	2	1.6	3	1.7	2	1.6	6	3.5		
	Taste aversion	18	12.6	38	20.8	18	12.6	36	19.5		
	High price	26	18.5	26	14	27	19	27	14.8		
Protein Supplements use	Yes	201	13.9	46	3.0	125	8.7	37	2.4	0.000	0.000

	**No**	1248	86.1	1476	97	1324	91.3	1485	97.6		
**Herbal Products use**	**Yes**	60	4.1	128	8.4	60	4.2	118	7.8	0.000	0.000
	**No**	1390	95.9	1404	92.2	1389	95.8	1404	92.2		
**Energy Drinks use**	**Yes**	350	24.1	170	11.2	239	16.5	118	7.8	0.000	0.000
	**No**	1099	75.9	1352	88.8	1210	83.5	1404	92.2		
**Source of Purchase**	**Pharmacy**	126	88.6	179	97.8	135	95	179	97.7	0.006	0.551
	**Supplements Store**	1	0.9	1	0.3	1	0.9	1	0.3		
	**Healthy Food Store**	1	0.4	0	0.3	1	0.4	0	0.3		
	**Gyms**	11	7.5	1	0.7	3	2.4	1	0.7		
	**Online Purchase**	4	2.6	2	1.0	2	1.3	2	1.1		
**Intake Form**	**Pills**	121	85	131	71.8	130	91.6	144	78.9	0.000	0.016
	**Capsules**	14	10	33	17.9	8	5.4	29	15.8		

	Others	7	5	19	10.4	4	3.0	10	5.3		
Read of DSs Label	Always	402	27.8	466	30.6	482	33.3	538	35.3	0.014	0.006
	Sometimes	414	28.6	495	32.5	429	29.6	496	32.6		
	Rarely	275	19.0	294	19.3	231	15.9	230	15.1		
	Never	358	24.7	267	17.5	308	21.2	259	17.0		

P1: significance value for male and female comparison before COVID-19

P2: significance value for male and female comparison during COVID-19

ANNEX 4: Estimated use of DSs

		Before COVID-19 Pandemic		During COVID-19 Pandemic		
		N	%	N	%	P-value
Multivitamins	Never	1809	60.9	1873	63	0.009
	Monthly	317	10.7	268	9	
	Weekly/Daily	846	28.5	831	28	
Antioxidants	Never	2345	78.9	2290	77	0.014
	Monthly	213	7.2	219	7.4	
	Weekly/Daily	415	14	464	15.6	
Vitamin A	Never	2255	75.9	2251	75.7	0.588
	Monthly	248	8.3	232	7.8	
	Weekly/Daily	469	15.8	489	16.5	
Vitamin C	Never	1362	45.8	1298	43.7	<0.001
	Monthly	561	18.9	423	14.2	
	Weekly/Daily	1049	35.3	1251	42.1	
Vitamin D	Never	1449	48.8	1400	47.1	<0.001
	Monthly	469	15.8	354	11.9	
	Weekly Daily	1054	35.5	1218	41	
Vitamin E	Never	2268	76.3	2232	75.1	0.002
	Monthly	252	8.5	218	7.3	
	Weekly/Daily	451	15.2	521	17.5	
Vitamin B12	Never	2061	69.3	2096	70.5	0.032
	Monthly	284	9.6	248	8.3	
	Weekly/Daily	627	21.1	628	21.1	
Folic acid	Never	2296	77.3	2279	76.7	0.365
	Monthly	214	7.2	213	7.2	
	Weekly/Daily	461	15.5	479	16.1	
Calcium	Never	2050	69	2104	70.8	<0.001

	**Monthly**	288	9.7	236	7.9	
	**Weekly/Daily**	634	21.3	632	21.3	
**Iron**	**Never**	1894	63.7	1974	66.4	<0.001
	**Monthly**	305	10.3	253	8.5	
	**Weekly, daily**	772	26	744	25	
**Magnesium**	**Never**	1826	61.4	1937	65.2	<0.001
	**Monthly**	347	11.7	258	8.7	
	**Weekly/Daily**	799	26.9	777	26.1	
**Phosphorus**	**Never**	2374	79.9	2355	79.3	<0.001
	**Monthly**	219	7.4	229	7.7	
	**Weekly/Daily**	378	12.7	387	12	
**Zinc**	**Never**	2177	73.3	1885	63.4	<0.001
	**Monthly**	235	7.9	215	7.2	
	**Weekly/Daily**	560	18.8	872	29.3	
**Other (Vitamins and minerals)**	**Never**	2604	87.6	2525	84.9	<0.001
	**Monthly**	100	3.4	124	4.2	
	**Weekly/Daily**	269	9	324	10.9	
**Protein supplements**	**Never**	2722	91.6	2811	94.6	<0.001
	**Monthly**	5	0.2	14	0.5	
	**Weekly/Daily**	244	8.2	146	4.9	
**Other protein supplements (Option 2)**	**Never**	2907	97.8	2937	98.8	<0.001
	**Monthly**	0	0	1	0.1	
	**Weekly/Daily**	65	2.2	34	1.1	
**Herbal products**	**Never**	2787	93.7	2793	94	0.035
	**Monthly**	14	0.5	4	0.1	
	**Weekly/Daily**	174	5.9	175	5.9	
**Other herbal products (Option 2)**	**Never**	2912	98	2923	98.4	0.129
	**Monthly**	6	0.2	1	0.0	
	**Weekly/Daily**	53	1.8	48	1.6	
**Energy drinks**	**< 7**	2696	99.9	2696	99.9	1
	**7 or above**	2	0.1	2	0.1	
